# Pregnancy, Microchimerism, and the Maternal Grandmother

**DOI:** 10.1371/journal.pone.0024101

**Published:** 2011-08-30

**Authors:** Hilary S. Gammill, Kristina M. Adams Waldorf, Tessa M. Aydelotte, Joëlle Lucas, Wendy M. Leisenring, Nathalie C. Lambert, J. Lee Nelson

**Affiliations:** 1 Division of Clinical Research, Fred Hutchinson Cancer Research Center, Seattle, Washington, United States of America; 2 Department of Obstetrics and Gynecology, University of Washington, Seattle, Washington, United States of America; 3 Inserm UMR639, Marseille, France; 4 Division of Rheumatology, University of Washington, Seattle, Washington, United States of America; Institut Jacques Monod, France

## Abstract

**Background:**

A woman of reproductive age often harbors a small number of foreign cells, referred to as microchimerism: a preexisting population of cells acquired during fetal life from her own mother, and newly acquired populations from her pregnancies. An intriguing question is whether the population of cells from her own mother can influence either maternal health during pregnancy and/or the next generation (grandchildren).

**Methodology/Principal Findings:**

Microchimerism from a woman's (i.e. proband's) own mother (mother-of-the-proband, MP) was studied in peripheral blood samples from women followed longitudinally during pregnancy who were confirmed to have uncomplicated obstetric outcomes. Women with preeclampsia were studied at the time of diagnosis and comparison made to women with healthy pregnancies matched for parity and gestational age. Participants and family members were HLA-genotyped for DRB1, DQA1, and DQB1 loci. An HLA polymorphism unique to the woman's mother was identified, and a panel of HLA-specific quantitative PCR assays was employed to identify and quantify microchimerism. Microchimerism from the MP was identified during normal, uncomplicated pregnancy, with a peak concentration in the third trimester. The likelihood of detection increased with advancing gestational age. For each advancing trimester, there was a 12.7-fold increase in the probability of detecting microchimerism relative to the prior trimester, 95% confidence intervals 3.2, 50.3, p<0.001. None of the women with preeclampsia, compared with 30% of matched healthy women, had microchimerism (p = 0.03).

**Conclusions/Significance:**

These results show that microchimerism from a woman's own mother is detectable in normal pregnancy and diminished in preeclampsia, supporting the previously unexplored hypothesis that MP microchimerism may be a marker reflecting healthy maternal adaptation to pregnancy.

## Introduction

Small numbers of maternal cells, acquired during fetal life, persist into adult life in healthy individuals as microchimerism [1], [2]. This microchimeric population of cells is partially genetically foreign to the person who harbors them, as the cells also express non-inherited maternal antigens (NIMA). Durable effects of NIMA have been described in later life tolerance to organ grafts as well as in susceptibility to some diseases [3], [4]. These observations raise the interesting question whether the microchimerism a woman harbors from her mother can impact either the course or outcome of her own pregnancies.

To our knowledge, microchimerism from a woman's own mother has not been studied during the course of pregnancy. We therefore conducted longitudinal studies of microchimerism from a woman's mother across gestational age in healthy pregnancies. To further explore a possible role for microchimerism from a woman's mother in maternal adaptation to pregnancy, we also sought to determine whether detection of microchimerism differed in the pregnancy complication preeclampsia, which is characterized by maternal immune dysfunction [5], [6].

Because a pregnant woman acquires another population of cells from the fetus during pregnancy [7], the current studies encompass three generations with more than one mother-child relationship. For clarity in discussion we will refer to the pregnant woman as the proband (P), to the mother of the pregnant woman as the mother of the proband (MP), and the child as the child of the proband, or fetus. Microchimerism from a woman's own mother will be referred to as MP microchimerism.

## Materials and Methods

### Ethics Statement

We obtained ethics approval for this study from the ethics committee at all institutions/hospitals where the participants were recruited and human experimentation was conducted, specifically the Institutional Review Boards of the Fred Hutchinson Cancer Research Center and the University of Washington. All individual participants gave written informed consent prior to participation in the study. For all children involved in the study, written informed parental consent was obtained. The form of assent/consent from the children depended on their age; for children aged 14 and years and older, written informed consent was obtained, for children aged 7–13 years, written assent was obtained, and for children less than 7 years old, verbal assent was obtained. This process was approved by the Institutional Review Board of the Fred Hutchinson Cancer Research Center, which was the only institution where children were recruited.

### Participants

We prospectively recruited healthy women with a singleton pregnancy seeking obstetric care in Seattle, Washington, USA between November 1995 and December 2008. Blood draws were requested from subjects during each trimester, and when available, preconception and postpartum.

After delivery, medical records and a self-reported questionnaire were collected and reviewed for clinical and demographic information. Information obtained included age, ethnicity, medical history, reproductive history, dating of the pregnancy, pregnancy complications, and maternal and fetal outcomes. History of transfusion was also queried. To fulfill our first objective, to describe the natural history of MP microchimerism over normal gestation, previously established exclusionary criteria were applied after delivery to restrict studies to women with a normal outcome. For the normal pregnancy group, we excluded subjects with the following obstetric complications: preterm birth (spontaneous or indicated), gestational hypertension, preeclampsia, diabetes, placenta previa, or placental abruption.

For the preeclampsia population, women were recruited at the time of clinical diagnosis at the University of Washington Medical Center. Preeclampsia was defined as hypertension (systolic blood pressure >140 or diastolic blood pressure >90) persistent for at least six hours with proteinuria (defined as a timed urine collection with ≥300 mg of protein in 24 hours, a random urine sample with a protein to creatinine ratio of ≥0·5, or a urine dipstick assessment of ≥3+). Severe preeclampsia was defined by the presence of any of the following: severe hypertension (systolic blood pressure >160 or diastolic blood pressure >110) persistent for at least six hours, seizures (eclampsia), hemolysis, elevated liver enzymes, thrombocytopenia, pulmonary edema, renal dysfunction, or fetal growth restriction [8], [9]. Subjects with chronic hypertension were included if a timed urine collection from early in pregnancy was available, and a twofold elevation in proteinuria was demonstrated in conjunction with worsening hypertension. All samples were drawn before the onset of labor. For the comparative analysis, participants with preeclampsia were matched one to one with healthy participants by gestational age and parity.

For all study probands, participation of the proband's mother was required for inclusion in the study, so that HLA-genotyping could be conducted to identify a polymorphism unique to the MP to target by HLA-specific quantitative PCR. Buccal swab sampling, mouthwash collection, or a blood draw was requested for this purpose. Fetal cord blood for genotyping was obtained immediately after delivery. Other family members were also invited to participate, including spouses/partners and any previously born children.

### Procedures

#### Isolation of Peripheral Blood Mononuclear Cells (PBMC) and DNA Extraction

Peripheral venous blood samples were drawn into acid citrate dextrose solution A-vacutainer tubes. PBMC were isolated from whole blood by Ficoll Histopaque (Pharmacia Biotech, Uppsala, Sweden) gradient centrifugation at a density of 1.077 g/ml. Genomic DNA was extracted from PBMC using Wizard Genomic DNA Purification Kits (Promega, Madison, WI) according to manufacturer's instructions. DNA was extracted from mouthwash specimens using the High Pure PCR Template Preparation Kit (Roche Diagnostics, Indianapolis, IN) or from buccal swabs using the BuccalAmp DNA Extraction Kit (Epicentre Biotechnologies, Wisconsin, USA).

#### Quantification of microchimerism by quantitative PCR

**Figure 1:**
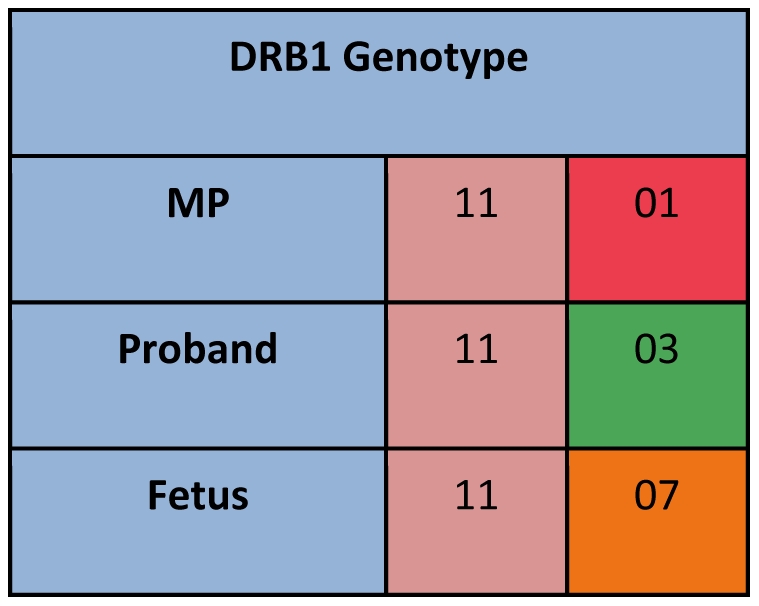
Schematic representation of strategy used to identify microchimerism. In this example, HLA genotyping for DRB1 is shown for a proband, her mother (MP), and her fetus. Once a polymorphism unique to the MP is identified (DRB1*01 in this example), polymorphism-specific quantitative PCR can then be used to quantify MP microchimerism in DNA extracted from proband PBMC.

Because of the extensive polymorphism in HLA genes, HLA genotyping of probands and their mothers usually results in identification of a polymorphism that is unique to the MP that can then be targeted to identify and quantify MP microchimerism. HLA genotyping was conducted using Dynal linestrips (prior to January 2008) or Luminex-based (One Lambda, Canoga Park, California, USA, beginning January 2008) PCR-sequence specific oligonucleotide probe techniques. All probands and family members were HLA-genotyped for the class II loci DRB1, DQA1, and DQB1. Familial HLA relationships were examined to identify non-shared HLA polymorphisms that could be used to uniquely identify MP microchimerism (for example, see Figure 1). In cases in which family HLA class II genotyping did not yield a testable polymorphism unique to the MP, other polymorphisms were typed including HLA-B and thyroglobulin deletion (Tg-deletion).

After identifying a polymorphism that was unique to the MP for each family, we employed the appropriate assay from a panel of HLA- and other polymorphism-specific qPCR assays that we developed for this purpose [10] to test DNA extracted from the proband's PBMC for MP microchimerism. Six to twelve aliquots of DNA from PBMC were tested from each blood draw, with total reaction volumes of 50 uL. The maximum amount of DNA tested per aliquot was 25,000 genome equivalents (gEq), as higher concentrations of DNA can inhibit the PCR reaction. A calibration curve for the polymorphism-specific assay was included to quantify the amount of MP microchimerism and validate the assay for each experiment. Every sample was also tested for a non-polymorphic gene, betaglobin (BGLOB). A BGLOB calibration curve (obtained from commercially prepared human genomic DNA [Promega]) was concurrently evaluated on each plate to quantify the total number of genome equivalents (gEq) of DNA tested in each reaction. DNA quantities were reported as the DNA gEq number of MP cells per 100,000 proband cell equivalents by using a conversion factor of 6.6 pg DNA per cell [11]. Total DNA tested per sample was considered acceptable if greater than 30,000 gEq.

Multiple safeguards were taken to avoid the risk of contamination. The optical detection system of the 7000 Sequence Detector obviates the need to reopen reaction tubes after amplification. DNA extractions and qPCR preparations were performed under an ultraviolet (UV) light equipped safety hood, with UV run for 30 minutes between experiments. Filtered tips were used during pipetting. Each experiment included control wells to ensure the absence of contamination.

### Statistical methods

Each sample obtained during and prior to pregnancy was described according to gestational age and trimester in pregnancy (pre-pregnancy, first trimester: <14 weeks, second trimester: 14–28 weeks, third trimester: >28 weeks) using the expected delivery date from the obstetrical provider's best clinical estimate. All subjects had sonographic confirmation of their dating during the first or second trimester.

Testing for microchimerism relies on stochastic sampling of an aliquot of proband cells (approximately 100,000 from each peripheral blood draw). The distribution of this type of data most closely approximates a Poisson process due to the rarity of microchimerism by definition. Therefore, to examine the impact of gestational age on changes in MP microchimerism levels in PBMC during normal pregnancy, we fit models utilizing a Poisson process for the rate of detection of MP microchimerism. This method models the probability of observing one positive cell (1 gEq) out of the total number of cells examined (therefore also accounting for any variations in total cell number tested). Due to the high degree of over-dispersion in the data, we utilized a negative-binomial model to obtain model estimates, with interpretation of results identical to that of a Poisson regression model. In the regression models, we analyzed increasing trends over the course of pregnancy, reporting an average incidence rate ratio (IRR) for change from one interval to the next (starting from prepregnancy and across subsequent trimesters or weeks). For the purposes of graphical presentation, a cubic spline function with 3 knots was fit as a function of weeks of pregnancy within the negative binomial model, and predicted rates calculated to illustrate the shape of the predicted rate of MP microchimerism over time.

For the analysis comparing MP microchimerism to subjects with preeclampsia, one control subject was identified for each preeclampsia case, matching on parity and gestational age. Overall detection of any MP microchimerism was compared using McNemar's test for matched pairs. Concentrations of MP microchimerism were compared using the Wilcoxon matched pairs signed rank test. All statistical analyses were conducted using Intercooled STATA 11·0 for Windows 2000 (StataCorp, College Station, Texas). All p values were 2-sided and considered significant at 0.05.

## Results

### Study participants and samples

Twenty-seven women with confirmed uncomplicated singleton pregnancies were included in the current study and analysis (Table 1). These women derived from an initial population of 55 healthy women who were enrolled and followed longitudinally throughout pregnancy, among whom complete family studies were obtained for 35 women. After performing HLA genotyping for all family members (n = 140), families were included when the proband did not develop any obstetric complications and the MP had a polymorphism that was unique and testable with our quantitative PCR panel.

**Table 1:** Clinical and demographic characteristics for study participants.

	Longitudinal Normal Pregnancy (n = 27)	Matched Normal Pregnancy (n = 20)*	Preeclampsia (n = 20)
Maternal age in years, mean ± SD	32·6±3·7	32·8±4·6	26·2±3·4
Nulliparity, n (%)	19 (70)	17 (85)	17 (85)
Gestational age at delivery in weeks, mean ± SD	40·3±1·3	40·2±1·4	32·7±3·8
Gestational age at sample collection in weeks, mean ± SD	n/a	33·2±3·9	32·2±3·8
Birthweight in grams, mean ± SD	3603±470	3517±538	1761±921
Small for gestational age (birthweight <10^th^ percentile), n (%)	2 (7)	2 (10)	13 (65)
Ethnicity, n (%)			
White	25 (93)	19 (95)	17 (85)
Other	2 (7)	1 (5)	3 (15)
Cesarean delivery, n (%)	5 (19)	4 (20)	12 (60)
Prior miscarriage, n (%)	3 (11)	2 (10)	7 (35)
Prior pregnancy termination, n (%)	3 (11)	1 (5)	3 (15)
Amniocentesis, n (%)	9 (35)	5 (25)	0 (0)
Chorionic villus sampling, n (%)	1 (4)	0 (0)	0 (0)
Male fetal sex, n (%)	14 (52)	10 (50)	9 (45)
Systolic BP in mm Hg, mean ± SD	n/a	n/a	162±25
Diastolic BP in mm Hg, mean ± SD	n/a	n/a	98±13
Renal dysfunction (Cr ≥1·0 mg/dL or oliguria), n (%)	n/a	n/a	5 (25%)
Hepatic dysfunction (AST ≥40 U/L), n (%)	n/a	n/a	4 (20%)
Thrombocytopenia (platelet count <100,000 cells/mm^3^), n (%)	n/a	n/a	1 (5%)
Overall severe preeclampsia	n/a	n/a	19 (95%)

*Comparisons between preeclampsia and normal pregnancy were made with a subgroup of normal pregnancy subjects matched for parity and gestational age.

The majority of women contributed two (n = 6), three (n = 12), four (n = 5), five (n = 2), or seven (n = 1) blood samples per pregnancy, with one participant contributing one blood sample. Pre-pregnancy blood draws were obtained up to eight months prior to pregnancy and postpartum blood draws up to one year after delivery.

For the preeclampsia group, 20 proband/MP pairs were included in the study and analyzed in comparison with 20 proband/MP matched pairs with normal pregnancy. Clinical and demographic information for the study population is provided in Table 1. Overall, the preeclampsia population reflected that of a tertiary care referral population, including 19/20 (95%) with severe disease.

No participant had a prior history of blood transfusion, and none was the recipient of an organ or hematopoietic cell transplant, including both the healthy participants and those with preeclampsia.

### Longitudinal MP Microchimerism in Normal Pregnancy

**Figure 2:**
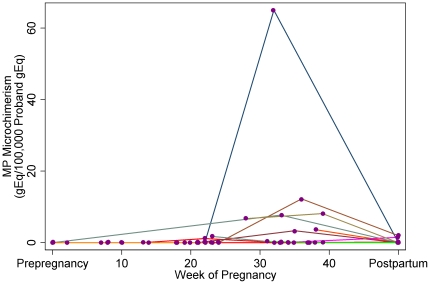
Concentration of MP microchimerism gEq in a stochastic sample of 100,000 proband gEq, among subjects with normal pregnancy, according to gestational age. Longitudinal datapoints for each subject (n = 27) indicated by colored lines.

We tested MP microchimerism in 86 PBMC samples from 27 women who were healthy and had uncomplicated pregnancies. MP microchimerism was identified in some women at some time points. The frequency of samples positive for MP microchimerism during each time interval was 0% (0/6) pre-pregnancy, 0% (0/9) in the first trimester, 16% (3/19) in the second trimester, 29% (7/24) in the third trimester, and 14% (4/28) postpartum. The concentration of MP microchimerism paralleled the overall frequency of detection (Figure 2). The ranges of MP microchimerism concentrations, expressed as MP microchimerism gEq per 100,000 proband gEq, for each time period: preconception, first trimester, second trimester, third trimester, and postpartum, respectively, were 0 - 0, 0 - 0, 0 – 6.7, 0 – 65.0, and 0 – 2.7. While many values were zero as expected, mean concentrations of MP microchimerism are also described as another window for quantitative trends in MP microchimerism concentration across gestation (though not statistically evaluated). The mean MP microchimerism concentrations (in MP microchimerism gEq per 100,000 proband gEq) were 0 pre-pregnancy, 0 in the first trimester, 5.1 in the second trimester, 41.8 in the third trimester, and 1.9 postpartum. The mean total proband gEq tested among the longitudinal normal pregnancy samples was 100,680 (standard deviation 26,028).

**Figure 3:**
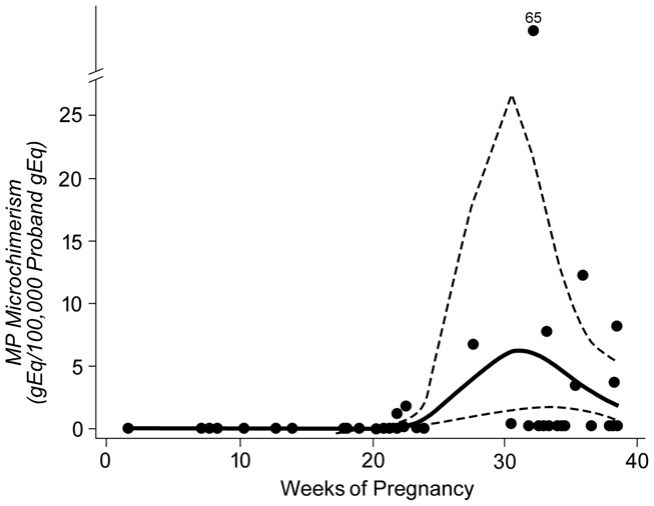
Curves indicate spline function showing model of expected concentration of MP microchimerism gEq in a stochastic sample of 100,000 proband gEq, according to gestational age. Solid line indicates the point estimate of expected microchimerism concentration, and dashed lines indicate the 95% confidence interval. Scatterplot shows MP microchimerism concentration in proband PBMC by trimester (in gEq per 100,000 proband gEq).

For each advancing time interval, including pre-pregnancy and each trimester of pregnancy, there was a 12.7-fold increase in the probability of detecting MP microchimerism relative to the prior interval [95% CI (3.2, 50.3); p<0.001]. For each additional week of gestational age (including pre-pregnancy as week 0), the probability increased 1.3-fold [95% CI (1.1, 1.5); p<0.001]. Visual representation of the expected concentration of MP microchimerism in a stochastic sample of 100,000 proband cells across gestation is shown as a spline in Figure 3.

### MP Microchimerism in Preeclampsia and Matched Controls

**Figure 4:**
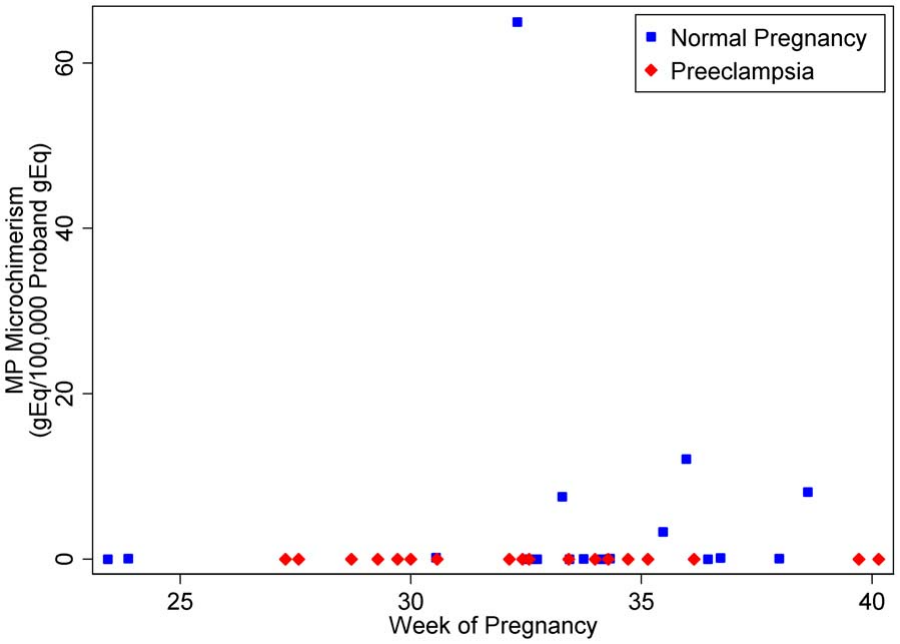
Concentration of MP microchimerism gEq in a stochastic sample of 100,000 proband gEq, among subjects with preeclampsia compared with subgroup of subjects with normal pregnancy matched for gestational age (range 24–40 weeks) and nulliparity (n = 40 total), according to gestational age.

Of the 20 probands with preeclampsia tested for MP microchimerism, none was positive (0/20, 0%). In comparison, in the matched group of women with healthy pregnancies, 6/20 (30%) were positive, p = 0.03. Quantitatively, the concentrations of MP microchimerism also differed significantly (p = 0.02), see Figure 4. The median MP microchimerism concentration for both groups was 0 GEq/100,000 host cells, as expected due to the skewed distribution. The MP microchimerism range for the matched normal pregnancy group was 0–65.0 gEq per 100,000 proband cell equivalents, with a mean concentration of 4.8 gEq per 100,000 proband cell equivalents. The mean totals of proband gEq tested among the preeclampsia samples and the matched control samples were 103,394 (standard deviation 30,373) and 100,124 (standard deviation 27,694), respectively.

## Discussion

It has only recently been appreciated that low levels of maternal cells, acquired during fetal life, persist into adult life in healthy immune competent individuals [2]. Although these cells could be a simple remnant of pregnancy without immunologic consequence, it is of interest to consider whether cells that a woman harbors from her own mother have the potential to influence a woman's pregnancies. To our knowledge, MP microchimerism has not previously been studied in reproductive health or during the course of normal or abnormal pregnancy. We investigated MP microchimerism in the peripheral blood of healthy pregnant women and found that, while MP microchimerism was not detectable early in gestation, it was not uncommon as gestation progressed, especially in the third trimester. We further conducted studies of women with preeclampsia, and in contrast to healthy pregnant women, no woman with preeclampsia had MP microchimerism in the third trimester. These results suggest that MP microchimerism could potentially influence pregnancy and/or be a marker reflecting healthy maternal immune adaptation during normal pregnancy.

Though a role for MP microchimerism in reproduction has not previously been explored, MP microchimerism has been shown to durably persist and to occupy diverse tissues and cell subsets with pluripotent capacity [12], [13], [14], [15]. In addition, pregnancy has been shown to alter the microchimerism harbored by a woman, with MP microchimerism declining in prevalence and concentration with increasing parity [16]. Classic experiments by Medawar in the 1950s showed that exposure to alloantigen in fetal life resulted in durable tolerance specific to that alloantigen [17]. That fetal exposure to MP microchimerism can result in clinically significant and durable tolerance in later reproductive life was suggested in studies that were reported in the 1950s. Owen and colleagues showed that the Rh status of a woman's mother was associated with her likelihood of developing alloimmunization during pregnancy. Specifically, if a woman's mother was Rh positive, the chance of developing erythroblastosis was significantly lower than if her mother was Rh negative [18]. This clinical observation supports the hypothesis that a woman's own in utero exposure to Rh positive cells conferred tolerance that prevented sensitization to Rh positive fetal cells during pregnancy.

In addition to reproductive outcomes, key evidence pointing to long-lasting effects of maternal cells acquired during fetal life comes from the transplantation literature. Clinical evidence suggests better outcomes with haploidentical solid organ [19] and hematopoietic stem-cell [20] transplants if matched to the non-inherited maternal HLA antigen (NIMA) as compared to donors matched to the non-inherited paternal HLA antigen (NIPA). This has been termed the NIMA effect. Recent studies have begun to delineate the functional role of MP microchimerism in its roles in the development of immunologic tolerance and in conferring the NIMA effect. Data show that maternal cells acquired by the fetus during pregnancy localize to fetal lymph nodes, where they cause proliferation of NIMA-specific fetal T regs [21]. The number and status of these NIMA-specific T regs correlates with MP microchimerism [22] and predicts transplant outcomes when NIMA-expressing allografts are subsequently transplanted [23]. The NIMA effect can also be conferred by infusion of NIMA-specific T regs into a non-tolerant animal [24]. The relationship between specific populations of T regs and tolerance of allogeneic cells is also supported by a recent study that showed colocalization of allogeneic HSC with T regs in bone marrow, associated with engraftment, with graft rejection resulting from selective elimination of T regs in the same model [25].

We have demonstrated an association of MP microchimerism with reproductive outcomes. Though conceptualizing a mechanistic framework for these associations is speculative, it is likely that MP microchimerism may become detectable in peripheral blood in normal pregnancies due to generalized stem cell mobilization, which increases with increasing gestational age. In normal pregnancy, potential triggers for such mobilization may be the load of fetal antigenic material seen in the third trimester [26], the increasing maternal blood volume and parallel need to increase circulating red blood cell mass [27], or the mobilization of endothelial progenitor cells seen later in gestation and that is likely important in maintenance of endothelial health [28]. Several possible explanations can be envisioned to underlie the relative deficit of MP microchimerism seen in women with preeclampsia. First, women with preeclampsia may have lower levels of MP microchimerism within stem cell populations due to an early deficit in acquisition of these cells during immune system development. Second, both women with normal pregnancies and those with preeclampsia may harbor MP microchimerism at similar concentrations, but women with preeclampsia may have a relative deficit in their ability to mobilize stem cells compared with women with normal pregnancies. A third possibility is that women with normal pregnancies and preeclampsia may both harbor and mobilize MP microchimerism equally well, but the women with preeclampsia may clear circulating cells more rapidly.

To our knowledge, this study provides the first description of MP microchimerism in pregnancy, characterized in a carefully defined longitudinal cohort of women with uncomplicated pregnancies and compared with a well-characterized group of women with preeclampsia. Our study limitations include feasibility issues limiting the number of participants investigated and the number of cells tested for each participant. We did not detect MP microchimerism in any of the women with preeclampsia. Statistically, we can say with 95% confidence that the probability of a random woman with preeclampsia having detectable microchimerism is less than 14%. This should not be interpreted to mean that our data rule out the possibility of detection of MP microchimerism in preeclampsia with a larger sample size, but rather that we found a statistically significant decrease in the overall detection and concentration in preeclampsia compared with normal pregnancy. We found that approximately one third of healthy women tested positively for MP microchimerism during pregnancy. It is likely that this is an underestimation of the true prevalence of microchimerism. Microchimerism, by definition, occurs at very low concentrations and may occur in any tissue. For practical reasons, we are able to test an aliquot of maternal peripheral blood mononuclear cells (∼100,000 cells), which means that we are not detecting microchimerism sequestered in bone marrow or other tissues. In addition, this stochastic sampling of a rare occurrence implies that microchimerism prevalence would be greater if larger amounts of maternal cells could be tested. In addition, some individuals may harbor microchimerism at concentrations below the lower limit (threshold) of detection of the assay, again leading to generalized underestimation. There is no reason to expect that this underestimation would vary by gestational age or across the groups of normal pregnancy and preeclampsia, thus rendering comparisons appropriate.

One additional point to consider is the lack of detectable MP microchimerism in women tested prior to pregnancy in this study. There were six subjects for whom preconception samples were available, and none of these six had detectable MP microchimerism. Given the limited number of subjects tested at this time point in the current study, it is unlikely that there is a statistically significant difference in this result compared with our lab's previous finding of 22% positivity in PBMC in healthy, nonpregnant women [10]. In contrast, we recently demonstrated 40% detection of microchimerism within granulocytes, also in healthy nonpregnant women [15]. Differences like this highlight the importance of consideration of specific cell population tested and underscore the possibility that pluripotent MP microchimerism is likely to be sequestered within the bone marrow or other tissues, rather than in peripheral circulation.

It is likely that the role of MP microchimerism in reproductive health is complex and depends on multiple factors. For MP microchimerism during pregnancy, multiple levels of interaction can be envisioned. The MP microchimerism can interact with the proband's cells and tissues, as well as with fetal microchimerism that is newly acquired during the pregnancy (which can also directly interact with the proband's cells and tissues). Our study suggests that interactions between a pregnant woman and her acquired microchimerism cell populations may have the potential to influence normal reproduction and that, when awry, these interactions may contribute to, or be a marker for, immune dysfunction in preeclampsia.
